# Monkeypox: A review of a zoonotic disease of global public health concern

**DOI:** 10.34172/hpp.2023.01

**Published:** 2023-04-30

**Authors:** Yusuf Amuda Tajudeen, Habeebullah Jayeola Oladipo, Abdulbasit Opeyemi Muili, Joy Ginika Ikebuaso

**Affiliations:** ^1^Department of Microbiology, Faculty of Life Sciences, University of Ilorin, P.M.B. 1515, Ilorin 240003, Nigeria; ^2^Department of Epidemiology and Medical Statistics, Faculty of Public Health, College of Medicine, University of Ibadan, P.M.B. 5017 G.P.O. Ibadan, Oyo State, Nigeria; ^3^Faculty of Pharmaceutical Sciences, University of Ilorin, P.M.B. 1515, Ilorin 240003, Nigeria; ^4^Faculty of Basic Medical Sciences, Ladoke Akintola University of Technology, P.M.B 4000, Ogbomosho, Oyo State, Nigeria; ^5^Department of Microbiology, Faculty of Natural Sciences, Chukwuemeka Odumegwu Ojukwu University, P.M.B. 02, Uli, Anambra, Nigeria

**Keywords:** Monkeypox, Global health, One Health, Zoonosis

## Abstract

**Background:** The rising circulation of the monkeypox virus while the COVID-19 is still ongoing in non-endemic countries is a significant global health threat. In this article, we have discussed the epidemiology, aetiology, and pathogenesis of the monkeypox virus to provide our current knowledge of the disease. Also, we discussed the ongoing efforts of the international health organizations to curtail the present epidemic and we finally provide recommendations for early detection and response.

**Methods:** We did a rapid literature search on PubMed, EMBASE, World Health Organization (WHO), the Centers for Disease Control and Prevention (CDC), and other trusted databases for recent articles (1958-2022) published in English—focusing on the outbreaks of monkeypox disease, epidemiology, pathogenesis, aetiology, prevention, and control in endemic and non-endemic countries. Keywords such as "Monkeypox", "Monkeypox virus", "Poxviridae", "Orthopoxvirus", "Smallpox", and "Smallpox Vaccine" were considered in our search based on MESH medical subject headings.

**Results:** Our review highlights four important findings. First, a cumulative of 1285 monkeypox cases have been documented and reported by the WHO in non-endemic countries as of June 8, 2022. Second, international travel contributes to the increase in cases in non-endemic countries. Third, the origin of the outbreak, the pattern of transmission, and the risk of infections is not fully understood. Fourth, there is an ongoing effort by the WHO, CDC, and other international health organization to control the spread of the monkeypox disease.

**Conclusion:** Our findings underline the need to reassess research priorities on the origin, transmission pattern, and risk factors for infection of monkeypox. Also, we provide recommendations under the One Health spectrum to prevent further spread of the disease.

## Introduction

 Monkeypox (Mpox) is a viral zoonotic infectious disease whose circulation has been previously limited to the African countries, specifically, the Central and West Africa. The causative agent of the disease is Mpox virus (MPXV)—a member of the genus *Orthopoxvirus* and the *Poxviridae* family.^1^ Several other viruses such as vaccinia and smallpox can also be found in this family—and these viruses have similar symptoms to that of the monkeypox.^2^ According to the disease history, the first isolation of the virus occured among the laboratory monkeys in 1958 in a Danish laboratory—where the name—Mpox—originated.^3,4^ In 1970, the Democratic Republic of the Congo reported the first human cases of the disease.^5^ The mode of transmission of the disease is through direct contact with infectious secretions from primates such as rodents and person-to-person transmission usually occurs through contact with infectious droplets of infected person.^6,7^

 Since the first reported case in human, the virus has been spreading sporadically in some African countries such as Nigeria, Ghana, the Democratic Republic of the Congo, Cameroon, Sierra Leone, Benin, Cote d’Ivoire, and Gabon.^8^ In 2003, the virus was reported outside its endemic region i.e. in the United States following the importation of infected rodents from Ghana to the city of Texas.^9,10^ Since then, the circulation of the disease have been reported in the non-endemic countries including the United Kingdom and Singapore.^9^ Increased human encroachment into animal reservoir habitat, rising international trade and travel, rapid population growth, and deforestation are some of the contributing factors responsible for the past outbreaks.^8,9^

 The World Health Organization (WHO), on the 7th of May 2022, announced a confirmed case of Mpox—which was reported from a traveller from Nigeria to the United Kingdom.^11,12^ In the face of the current epidemic which is believed to have been caused by the West Africa clade, between May 13, 2022, and June 8, 2022, a cumulative of 1285 confirmed cases of MPXV have been reported in 28 non-endemic countries.^13^ These confirmed cases have surpassed the total number of cases reported in non-endemic countries since 2003. Even though no known cases of death reported, there is still a knowledge and research gap about the origin, transmission pattern, and risk factors for infection. However, while epidemiological investigations are still ongoing, there is a high possibility of detecting more cases in countries where the disease is not endemic i.e. non-endemic countries. In this article, we have discussed our current scientific knowledge of epidemiology, aetiology, and pathogenesis of MPXV. Also, we discussed the ongoing measures put in place by international health organizations like the WHO and the Centers for Disease Control and Prevention (CDC) to curtail the present epidemic and we finally provide recommendations for early detection and response.

## Materials and Methods

 We conducted a comprehensive narrative review of recent and relevant search of literature published in English from 1958 to 2022. During our search, we also considered relevant databases and traditional search engines using information from relevant international health organizations, CDC, PubMed, Google Scholar, Science Direct, as well as national institutes of research on Mpox in endemic and non-endemic countries. During our search, keywords such as “Monkeypox”, “Monkeypox virus”, “Poxviridae”, “Orthopoxvirus”, “Smallpox”, and “Smallpox Vaccine” were considered in our search based on MESH medical subject headings. Reference lists of downloaded articles were thoroughly checked manually for additional titles included in the article reviewed. Reports on Mpox without public health focus were excluded. All articles were screened for eligibility and relevant articles were considered in the final review if they supported the aims and objectives of this research. Articles included in the final review were published between 1958 and 2022 and contained information on the outbreak, epidemiology, pathogenesis, aetiology, and public health interventions on Mpox. Epidemiological and clinical studies finally reviewed and included in our study reported the trends and health threat of the disease in both endemic and non-endemic countries as well as the ongoing interventions from the public health perspectives. Information on these important aspects of Mpox are extracted from the screened literature while the findings are presented in the result section below.

## Results

 Our review highlights four important findings. First, a cumulative of 1285 Mpox cases have been documented and reported by the WHO in non-endemic countries as of June 8, 2022. Second, international travel contributes to the increase in cases in non-endemic countries. Third, the origin of the outbreak, the pattern of transmission, and the risk of infections is not fully understood. Fourth, there is an ongoing effort by the WHO, CDC, and other international health organization to control the spread of the Mpox disease.

## Brief history of Mpox virus

 Mpox is indigenous to the rainforests of Africa i.e. the Central and West Africa.^14^ Indirect exposure to the disease may be more frequent in people living in proximity to the forested areas and this could lead to subclinical infection thereby spreading the disease unknowingly in the population. People living in this region have high vulnerability to the disease.^15^ In August 1970, when the incidence of smallpox was eradicated, in a small village in the Democratic Republic of the Congo, the first human case of the disease was reported. The case was a 9 month old child—who was suspected of smallpox virus in the Basankusu hospital.^16^ However, the confirmatory result of the patient sample from the WHO Small Reference Centre in Moscow revealed the presence of the MPXV.^16^ There are basically two genetic clades of the MPXV i.e. the Central African clade otherwise known as the Congo Basin clade and the West African clade. Of these two clades, the Congo Basin has high transmission rate and has also been reported to account for more severe cases of MPXV when compared to the West African clade.^16,17^ Also, this clade is responsible for the outbreaks occurring in the Central African countries, the virulent level is high and with 10.6% fatality rate.^10^ Comparingly, the West African clade is less virulent with a 3.6% fatality rate and it is responsible for the outbreaks occurring in the West African countries and those reported outside Africa.^2,10^ These two clades have been reportedly found in Cameroon.^10^ Over the years, the suspected reservoir hosts for the MPXV include but not limited to squirrels, dormice, and Gambian Pouched rats.^18^

## Transmission

 Transmission of the disease from animals to humans occur primarily through direct contact with animal mucosal surfaces either through bites or scratches obtained from infected animals—harboring the virus. Even though the virus has been isolated from small mammals and the nonhuman primates, a known definitive host has not been confirmed. In an instance where the virus is isolated from reservoir animal host, specifically the wild animal, there is always an appearance of lesions—which is an indication of active infection. However, due to poor surveillance and little serological surveys in nonhuman primates and wildlife species, it is uncertain whether there are asymptomatic carriers in animal species.^7,18,19^ However, a study conducted by Doty and colleagues in the Democratic Republic of the Congo has revealed the circulation of *Orthopoxvirus* in some wild animal species.^20,21^ The virus can also be transmitted through the consumption of undercooked or poorly cooked bushmeat. Large respiratory droplets can be transmitted from person to person during prolonged face-to-face contact, and congenital Mpox infection can also be transmitted through the placenta.^18,19^ To the best of our knowledge, the ongoing outbreaks of Mpox is not as a result of animal-to-human contact but rather due to contact from person-to-person.

 Person-to-person transmission can also occur and this is usually via direct contact with infectious secretions (such as lesions and respiratory droplets) of infected individuals, contact with materials (such as beddings and clothing) contaminated with bodily fluids or sores of an infected patient.^7^

## Epidemiology of Mpox

###  Previous outbreak

 From 1981-1986, an estimated 338 cases of Mpox was documented in the Democratic Republic of the Congo in a total population of 5 million and in an outbreak that occur from 1996-1997—the proportion of an at-risk population that contracted the disease was placed at an attack rate of 22 cases per 1000 population of people.^22^ In Democratic Republic of the Congo, the emergence of Mpox continues to occur drastically while sporadic occurrence continue to be reported in the neighbouring countries.^22^ From 1970-2018, countries including Nigeria, Cameroon, Democratic Republic of the Congo, Côte d’Ivoire, Nigeria, Sudan, and Sierra Leone have documented cases of Mpox.^22,23^ Even though the virus was initially thought to be geographically limited to the Central and West African countries, the 2003 outbreak reported in the United States of America is a clear evidence that the virus is not endemic only to Africa countries.^9,24^

###  Recent and ongoing outbreak 

 Prior to the 2022 ongoing outbreak, the United Kingdom have reported cases of Mpox between May 25, 2021, and June 15, 2021.^12^ The first case was from a traveler from the West Africa country i.e. Nigeria on May 8, 2021.^2^ In 2022, the disease has now become a global burden in addition to the cases of COVID-19, Malaria, polio, and influenza. On May 7, 2022, Mpox cases were reported in European countries such as United Kingdom and Spain including other countries.^11^ According to the WHO epidemiological report on the MPXV, 24 non-endemic countries have reported cases (suspected and confirmed) of MPXV, and some of these cases neither have a travel history to United kingdom, Spain nor the Western Europe.^25^ In another epidemiological report of June 5, 2022; 920 confirmed cases of Mpox and 70 Mpox suspected cases have been made known and reported.^25^ 64 of these confirmed cases have a known travel history link with countries such as Europe (32 cases), Canada (2 cases), West Africa (3 cases), Australia (1 case), and the travel history location of 26 cases remain under epidemiological investigation.^25^ In the latest situation report update of the WHO and as of June 8, 2022, the confirmed cases have increased to 1285 from an estimated 28 non-endemic countries (Table 1).^13^ Confirmed cases are mostly from the European region—where a cumulative 1112 cases have been reported.^13^ The Region of the Americas have reported 153 cases, the Eastern Mediterranean Region have reported 14 cases, and Western Pacific Region have reported only 6 cases.^13^ Cases reported are mostly in men within the age bracket 20-50, with rising cases in the MSM community i.e. men who have sex with men.^6^ Importantly, Mpox is not transmitted sexually, hence, its circulation in the MSM community is a concerning issue that needs to be investigated to have a better knowledge of the origin of the outbreaks and factors facilitating the risk of infections.^6^

**Table 1:** confirmed cases of monkeypox in non-endemic countries as of June 8, 2022^13^

	**Country**	**Confirmed cases**
Europe	United Kingdom	321
	Spain	259
	Portugal	191
	Germany	113
	France	66
	The Netherlands	54
	Italy	29
	Belgium	24
	Switzerland	12
	Ireland	9
	Slovenia	6
	Sweden	6
	Czechia	6
	Denmark	3
	Finland	3
	Israel	2
	Latvia	2
	Hungary	2
	Norway	2
	Malta	1
	Austria	1
Americas	Canada	110
	United States of America	40
	Argentina	2
	Mexico	1
Eastern Mediterranean	United Arab Emirates	13
	Morocco	1
Western Pacific	Australia	6
Cumulative	28	1285

## Aetiology of Mpox

 MPXV is a double-stranded DNA, oval brick-shaped of the family *Poxviridae *and, subfamily *Chordopoxvirinae*. Other members of the family include Variola virus, Vaccinia virus (the virus used in smallpox vaccination), Cowpox virus, Camel pox virus, and Ectromelia virus.^26^ Since smallpox eradication, Mpox has become the most common *Orthopoxvirus* to affect humans.^27^ MPXV is around 200-250nm in size, with a dumbbell-shaped core and distinctive surface tubules. The genome of the virus is about 199 kb of double-stranded linear DNA and about 190 Open reading frames > 180 nm long that are non-overlapping.^28-30^

 Certain risk factors influence the occurrence of Mpox infection in humans. The discontinuation of the smallpox vaccination campaign is the most important risk factors, and this has resulted in waning cross-protective immunity,^31^ making the younger age group particularly vulnerable to the infection.^1^ The increased direct interaction between humans and the virus’s reservoir hosts in the hotspot zone is another factor.^31^ As a result; the MPXV has the ability to infect considerably large numbers of small mammals acting as the reservoir hosts. Some of these reservoir hosts include Nesomyid rodents (*Cynomys *sp.,*Funisciurus*sp.,*Graphiurus*sp.*, Cricetomys*sp.), Sciurid, Glirid, and marsupials (*Monodelphis domestica, Didelphis marsupialis*).^20^ Specifically, the re-emergence of human Mpox has been facilitated by rural dwellers’ consumption of bush foods, climatic change, population movement, rainforest exploitation, declining herd immunity as well as other factors like transboundary migration, and issues of geopolitical conflicts in endemic areas.^22,32,33^

**Figure 1:**
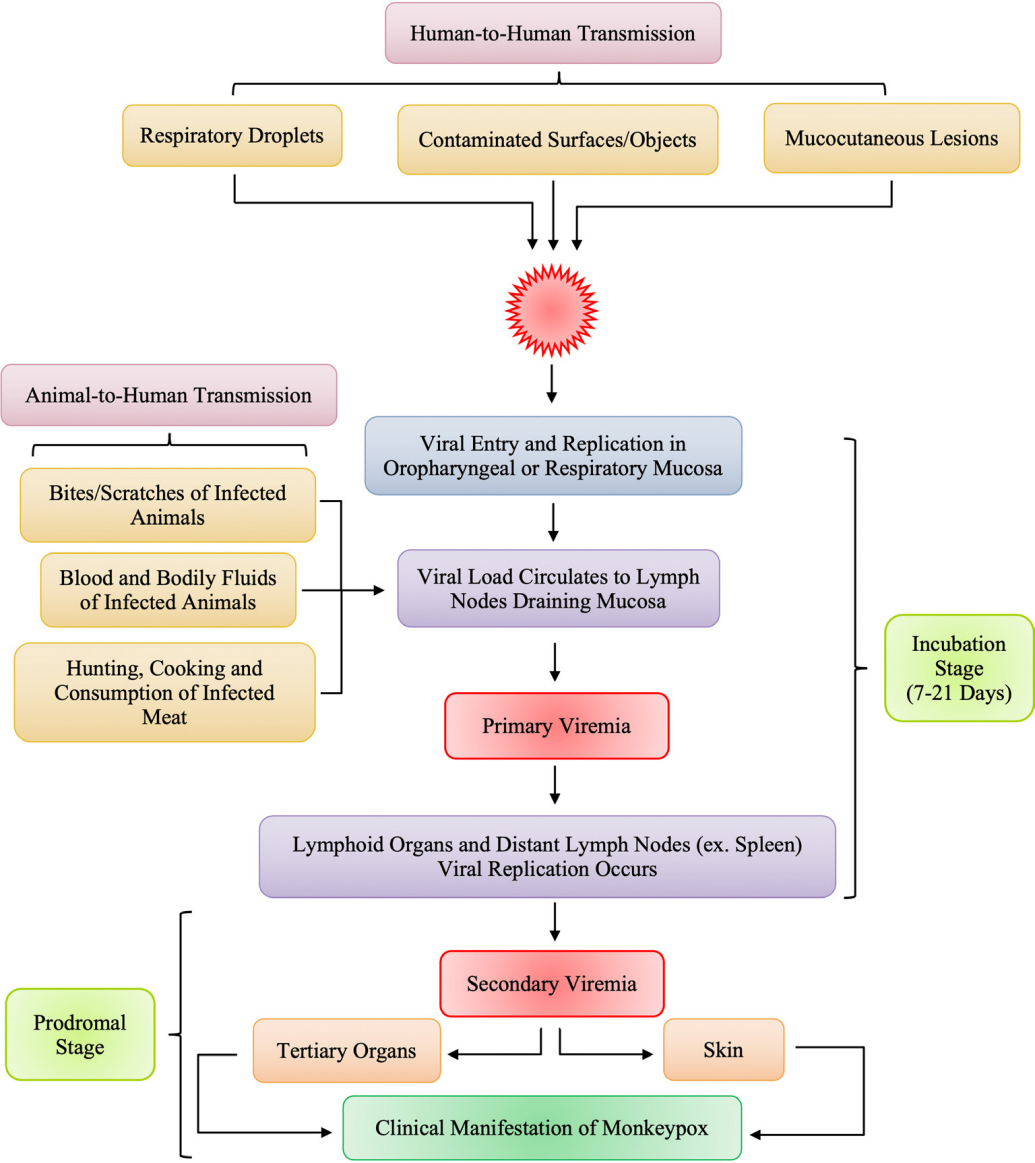
Pathogenesis of Monkeypox in Humans^34^

 The human Mpox has a very similar clinical presentation to that of distinct, typical smallpox with both having an incubation period of 7 to 14 days. There is an initial feverish prodromal stage that occur between 1 to 4 days, and this is followed by a rash duration that lasted between 14-21 days (Figure 1).^34^ Similarity also occur in the appearance in the appearance, distribution, and progression of lesions in MPXV and smallpox^27^ with symptoms such as fever, backache, myalgia, and lymphadenopathy. These are usually followed by well-circumscribed rashes that is generalized coupled with the development of centrifugal pattern through the macular, papular, vesicular, and the pustular stages.^35,36^ The condition of the patient becomes deteriorated by the appearance of the second febrile period marked by pustular lessions.^37^ There is a more severe disease—mostly associated with pronounced illness, high presence of the virus in the blood, and finally death, without a sustained infection following direct human-to-human transmission.^8,16,38^ Vaccination against smallpox provides considerable protection, with unvaccinated people experiencing 74% more severe consequences than vaccinated people (39.5%).^27^

## Pathogenesis of Mpox

 The outbreaks of Mpox are prevalent among the rural dwellers that engage in ecosystem activities such as wildlife hunting. Their close physical contact is the most significant risk factor for infection.^39^

 The pathogenesis of Mpox infection starts with viral entry through the skin cells or respiratory mucosa, depending on the mode of transmission from wildlife source or person to person contact. The virus replicates at the initial inoculation site (skin or respiratory tract) and then spreads to the local lymph node drainage, causing primary viremia. The MPXV then replicates in lymphoid tissues, infecting other lymphatic organs such as the spleen. The virus is subsequently released into the systemic circulation, causing secondary viremia. Once in the blood stream, MPXV localizes again in skin cells or respiratory mucosa causing skin lesions and viral spread in saliva and other bodily secretions.^40,41^ However, the host immune response plays a vital role, as other factors such as the duration of contact with infected animal or person and the viral density in bodily secretions and skin lesions, and virus virulence. Thus, the risk of transmission largely depends on MPXV evasion mechanisms and the host immune response.^41^

## Ongoing efforts for Mpox control

###  Preventive and control measures

 Mpox has re-emerged with a wider spread making it a slow-rising pandemic and no longer an African endemic disease. Several measures have been put in place to mitigate the widespread and ensure proper management of this infectious disease. These measures will be viewed from the preventive and current treatment angle recommended by the CDC.

###  Preventive measures

 The transmission of the MPXV can be reduced by the following preventive measures.^42^

- Infected patients that may be at risk of infection should be isolated.
- The use of standard personal protective equipment (PPE) when caring for suspected or declared cases of Mpox viral infection in humans or animals should be encouraged.
- Discourage contact with animals (dead or alive) that harbor and serve as carriers of this virus, especially in areas with recorded cases.
- Avoid direct contact transmission through infected materials that must have been used by an infected human or animal.
- Promotion and practice of good personal hygiene such as hand washing, especially after contact with suspected humans or animals is encouraged.

Additionally, scientist alongside the U.S. Food and Drug Administration (FDA) has approved the use of JYNNEOS^TM^ (also called Imvamune or Imvanex)—an attenuated live virus vaccine that has the potential of serving as pre-exposure prophylaxis together with the conscious measures taken to prevent the spread of the virus.^42^

###  Treatment

 As of the publication of this article, there are no clinically approved treatments for the Mpox viral infection. However, the treatment used for the smallpox virus may prove beneficial as they belong to the same genus. There are several countermeasures from the Strategic National Stockpile (SNS) that serve as treatment options for the MPXV.^42^ They include:

- Tecovirimat (also called TPOXX) is an antiviral medication readily available in pill or injection form and was originally used for the treatment of smallpox in humans.
- Cidofovir (also called Vistide) is an approved antiviral drug that can be used for the treatment of infections from *Orthopoxviruses* especially during an outbreak.
- Brincidofovir (also called Tembexa) is a recently approved drug by FDA that is yet to be made available from the SNS. It is initially used for the treatment of smallpox in humans but the EA-IND is being developed by the CDC to facilitate its use for the treatment of monkeypox.
- Vaccinia immune globulin intravenous (VIGIV) is an FDA-approved antiviral drug known initially for the treatment of vaccinia viral infections. However, an expanded access protocol has been hold by the CDC—and this protocol permits the use of stockpiled VIGIV for *Orthopoxviruses* treatment in case of an outbreak.

It will be worthy to note that most of these countermeasures are placed under “compassionate use” as per the CDC protocol.^42^

###  Efforts by international health authorities

 Mpox outbreak in the non-endemic countries is quite concerning and has generated increased attention from several international health authorities such as the WHO, the Food and Agricultural Organization (FAO), CDC, and International Atomic Energy Agency (IAEA). These organizations have embarked on several ongoing efforts for early detection, response, and control of the current epidemic of Mpox.

 Part of the ongoing efforts is evidenced by the IAEA partnership with other international organizations and the ZODIAC (ZODIAC “Zoonotic Diseases Integrated Action” is an initiative launched during the COVID-19 pandemic for early detection and control of zoonotic diseases) national laboratories in some part of the world, specifically, Africa, Asia, Europe, and the Latin America to make use of nuclear science and technology to facilitate the early detection and rapid diagnostic algorithm for the Mpox disease.^43^ This is to have a comprehensive understanding of how the virus circulates in the non-human host, its survival in the environment, and species-to-species transmission since there is a scarcity of data on this aspect.^43^ Also, the IAEA Centre for Nuclear Techniques in Food and Agriculture through their joint action with the FAO i.e. joint FAO/IAEA is working towards facilitating the development of detection tools to understand the importance of rodents as well as other small mammals in zoonotic infections of Mpox.^43^

 Following the meeting convened by the WHO in response to investigating the cause of the outbreak and minimizing further spread, the organization published its interim guidance on surveillance, case investigation, and contact tracing in May 22, 2022.^44^ It also advises the Member States to strengthen collaboration with the ongoing investigation and shared with them the surveillance guidance in the Epidemiological Alert on Mpox in the regions where the disease is not usually spread.^44^ This is to allow for prompt information collection for clinical and epidemiological characterization of cases. Interim guidelines on laboratory testing for Mpox were also shared with the Member States.^45^ Laboratory testing kits including polymerase chain reaction (PCR) were mobilized for rapid detection of cases. Through this, an estimated 13 European countries have sequenced the genome of the MPXV, including the United States of America.^13^ In June 24, 2022, the WHO updated its surveillance, case investigation, and contact tracing to apply the interim guidance to all countries.^46^ This updated guidance include countries where the virus is historically endemic, countries where the virus is currently circulating, countries with potential cases, and countries which have not documented the transmission of the disease. This aimed at reducing the human-to-human transmissions of the disease and to prevent further outbreak which has been linked to growing international travels outside Africa.^46^

 Just as at the time of publication of the interim guidelines of May 22, 2022, Kraemer and colleagues supported a global response efforts by creating an open-access database and visualization aimed at tracking Mpox cases across different countries.^25^ This database that was developed in line with the metadata (such as sex, age, laboratory confirmation, travel history, and symptoms) of the WHO will allow for a better understanding of the origins of the outbreaks and transmission dynamics. These researchers point out the challenges associated with epidemiological data reliability and synthesis on characteristics of cases at the early stages of the outbreaks, and recommend the need for real-time data to halt the transmission progress of Mpox.^25^

 Consequently, the WHO has developed surveillance case definitions (available for suspected cases, probable cases, confirmed cases, and discarded cases) to enhance easy identification through distinguishable symptoms.^7^ The WHO is also working with the Member States in African countries and non-endemic countries, regional institutions, and technical and financial partners to reinforce the effort and allow for rapid and efficient laboratory diagnosis of MPXV. For example, the use of PCR tests has allowed for rapid detection of Mpox DNA in patients’ tissue and confirmation of suspected cases is achieved through enzyme-linked immunosorbent assay (ELIZA), Immunofluorescence Assay, and tissue culture.^7,24,26,44^

 Additional support also includes expansion of disease surveillance in non-endemic countries, readiness, and prompt response actions to prevent the further spread of the Mpox. Beyond this, the WHO is also providing expertise to public health workers through technical and epidemiological guidance on contact tracing, testing, clinical management, isolation, prevention, and control measures for the Mpox diseases in both endemic and non-endemic countries.^47,48^

 Currently, two types of vaccines (i.e. ACAM-2000 and Modified Vaccinia Ankara, MVA-BN) used against Mpox have been deployed by some Member States.^13^ For example, MVA-BN, a smallpox vaccine shown to be protective against Mpox, has been approved for use in countries such as Canada and the United States of America for the prevention of close contact.^13^ However, the experts convened by the WHO are currently reviewing the latest developments on these vaccines to provide appropriate guidance for use.

 The antiviral drug for *Orthopoxviruses* i.e. tecovirimat, has also been approved by the United States Food and Drug Administration, European Medicines Agency, and Health Canada to treat smallpox infection only.^13^ However, it is not approved by the FDA to be used for other *Orthopoxviruses* infections of which Mpox is inclusive. However, the CDC held expanded access to Investigational New Drug (EA-IND)^49^ and this protocol permits early empirical treatment of Mpox as well as other Orthopoxviruses infections using tecovirimat.

 Since awareness plays an important role in ensuring prevention, early detection, targeted therapy, and effective treatment, WHO is educating the public about the Mpox, vaccines, and immunization, as well as risk communications and providing support for the Member States in raising awareness.^13^ The WHO is also offering expertise and training for public health workers on how to collaborate with local communities in a bid to eradicate Mpox disease.

## The One Health approach

 The One Health approach was introduced in the 1800s in a bid to address the rising effect of the health crisis which occurs at the interface between humans, animals, and their environments.^50^ It is one of the oldest approaches to health and the observation of the similarities in disease processes between humans and animals prompted its introduction by the scientists.^50^ Since the advancement of the approach in the 20th century towards addressing the emerging pandemic threat of diseases such as severe acute respiratory syndrome,^51^ it has continued to gain increased attention from academic researchers and international health partners to address the public health threats at human-animal nexus. The approach is very effective in that it involves multidisciplinary collaboration and combined efforts of concerned individuals working at local, national, and international levels to achieve a common goal of optimizing the health of humans, animals, and environments.

## Recommendations through the lens of One Health

 Since Mpox is a viral zoonotic infectious disease occurring at the interface between human, animal, and the environment, the One Health approach needs to be adopted to address the ongoing crisis and to effectively minimize the occurrence of such outbreaks in the future. What are the specific strategies we need to develop under the One Health spectrum to effectively control the spread of Mpox? First, there is an urgent need for the development and deployment of unified integrated global zoonotic disease surveillance systems in order to generate and covey required data to inform evidence-based responses, thereby minimizing the cross-border transmission risk of Mpox. Second, the origin of the outbreaks, risk factors for infection, and pattern of transmission are not well understood. Hence, scientists including epidemiologists, public health scientists, microbiologists, veterinarians, virologists, and molecular biologists must lead collaborative research programs supported by adequate research funding from government and international donors to better understand the epidemiological trend of the disease. Third, well-equipped laboratories with molecular diagnostic tools for rapid sequencing of the viral DNA are needed in endemic countries, to enhance the early detection of cases and prevent further spread. This is because only a few laboratories are equipped with the required diagnostic tools in endemic countries. Fourth, wildlife trade plays a prominent role in the transmission of Mpox, a targeted ban on trading of wildlife that poses a public health risk should be implemented as soon as possible. Fifth, under the One Health approach, a committee of international experts should be convened and charged with the duties of reviewing the public health measures such as treatment options and safety protocols, and containment strategies put in place for Mpox. Sixth, community healthcare systems should be improved and healthcare resources should be allocated to regions where the disease is endemic to enhance response and control. Seventh, community-based research should be prioritized in endemic countries to understand the perception of the local communities, risky attitudes, and health-seeking behavior on Mpox. Eighth, constant person-to-person awareness about the transmission and Mpox safety measures is required to prevent the further spread of the disease, especially in the rural areas with poor access to media devices and digital communication tools.

## Conclusion

 The outbreak of Mpox amidst the COVID-19 pandemic revealed the fact that human vulnerability to zoonotic infectious diseases is considerably high. The unavoidable international mobility is facilitating cross-boundary transmission of pathogens of public health significance which continue to adversely affect humans. An important lesson from the ongoing COVID-19 pandemic was that early prevention and response are important factors to consider in disease containment. While the WHO has announced Mpox as a public health emergency of international concern and the transmission is higher in the MSM community, raising awareness to deal with challenges including stigmatization and homophobia combined with strengthening public health preparedness and response effort in terms of diagnostic and preventative capacity is an important step to curtail the epidemic from further spread.

## Acknowledgements

 Special thanks to Dr Mona Said El-Sherbini, an associate professor at the faculty of Medicine, Cairo University, Giza, Egypt, for her comments and suggestions towards improving the quality of this manuscript.

## Competing Interests

 The authors declare no competing interest.

## Ethical Approval

 Not required.

## Funding

 Authors have received no funding for the conduct of this research
